# Enhanced silk production and pupal weight in *Bombyx mori* through CRISPR/Cas9-mediated circadian *Clock* gene disruption

**DOI:** 10.1371/journal.pone.0317572

**Published:** 2025-01-27

**Authors:** Daniel J. Brady, Alessio Saviane, Matteo Battistolli, Irene Varponi, Federica Barca, Kunihiro Shiomi, Silvia Cappellozza, Federica Sandrelli

**Affiliations:** 1 Department of Biology, University of Padova, Padova, Italy; 2 Fraunhofer Institute for Molecular Biology and Applied Ecology—IME, Branch for Bioresources, Schmallenberg, Germany; 3 Council for Agricultural Research and Economics, Research Centre for Agriculture and Environment, Sericulture Laboratory of Padova, Padova, Italy; 4 Faculty of Textile Science and Technology, Shinshu University, Matsumoto, Japan; USDA Agricultural Research Service, UNITED STATES OF AMERICA

## Abstract

The domesticated silkworm, *Bombyx mori*, is crucial for global silk production, which is a significant economic activity supporting millions of livelihoods worldwide. Beyond traditional silk production, the growing demand for insect larvae in cosmetics, biomedical products, and animal feed underscores the need to enhance *B*. *mori* productivity. This study investigates the role of the circadian clock gene *Clock* in *B*. *mori* using CRISPR/Cas9-mediated mutagenesis to establish the *Clk*^*Δ29*^ knock-out mutant strain. Dysregulation of the circadian clock in *Clk*^*Δ29*^ was demonstrated by altered temporal transcriptional profiles of core circadian clock genes in adult heads and disrupted circadian-controlled behaviors, including adult eclosion and egg hatching rhythms under constant darkness. By analysing larval development timing, as well as the weights of late instar larvae, pupae, and cocoon components in *Clk*^*Δ29*^ mutants and in *Clk*^*Δ1922*^ silkworms (carrying an independently generated *Clk*^*-*^ null allele), we showed that CLK contributes to physiological processes regulating *B*. *mori* development and growth. Importantly, *Clk*^*Δ29*^ mutants reared on a standard sericulture diet exhibited significant increases in key economic traits, with silk production increasing by up to 7%, and pupal weight increasing by up to 25% compared to wild-type controls. This study highlights the potential of circadian clock gene manipulation to significantly enhance sericultural productivity. Future research should focus on elucidating the molecular mechanisms driving these phenotypes and determining whether they result from circadian clock functions or pleiotropic effects of *B*. *mori Clk*. These findings provide a foundation for advancing sustainable sericulture and developing new commercial applications for silkworm-derived products.

## Introduction

Sericulture, the cultivation of silkworms for silk production, is a significant economic activity that provides substantial employment and supplementary income for millions worldwide, particularly in China and India [1]. The mulberry silkworm, *Bombyx mori*, is the primary species used for silk production globally, underlining its critical economic role. In recent years, the use of insects and their byproducts in cosmetics, biomedical products, and animal feed has also received considerable attention [2, 3]. In 2021, the European Commission approved the use of *B*. *mori*-derived proteins for animal feed, and in 2024, the first commercial pet food featuring silkworm proteins was released on the European market [4].

Traditional genetic breeding methods have led to a massive diversification of silkworm lines and qualities, significantly contributing to the sericulture industry. However, modern biotechnology, particularly gene editing technology, holds the potential to revolutionise this field by drastically improving efficiency. By modifying the expression of genes related to silk gland development, silk protein synthesis, and other physiological characteristics, researchers can rapidly develop silkworm strains with desirable traits, paving the way for unprecedented advancements in silk production. Given the economic importance of silk production and the growing demand for high-quality silkworms, enhancing sericultural outputs is essential for maintaining economic sustainability and growth in the sector. Necessitating continued improvements in silkworm productivity and quality through better breeding practices, optimised rearing conditions, and genetic enhancements.

Recent studies have begun to explore site-specific and targeted molecular genetic manipulation as a means to enhance silkworm productivity [5, 6]. Continued innovative methods are critical for achieving the necessary improvements in silk yield and overall silkworm performance, ensuring the future success and sustainability of the sericulture industry.

One promising area of interest is the circadian clock, an internal timekeeping mechanism that regulates gene expression and numerous physiological and behavioural processes throughout the day. The circadian clock synchronises (entrains) physiological and behavioural activities with environmental cues, optimising biological functions and enhancing fitness [7]. In Lepidoptera, circadian rhythms influence egg hatching, adult eclosion, feeding, and metabolism, all of which are vital for their ecological roles and economic value [8–10].

The first molecular model of an insect circadian clock was developed in *Drosophila melanogaster* and features interlocking autoregulatory transcription-translation feedback loops (TFLs) that generate the rhythmic oscillation of core clock components [7, 11]. The main TFL consists of the proteins: CLOCK (CLK), CYCLE (CYC), PERIOD (PER), TIMELESS (TIM) and CRYPTOCHROME (CRY). The so-called positive arm of the clock comprises CLK and CYC, transcriptional factors that dimerise via CLK’s Per-Arnt-Sim (PAS) domains, bind to DNA E-box elements via their basic Helix-Loop-Helix (bHLH) domain and promote the expression of *per* and *tim* transcripts. Later, PER and TIM proteins (forming the negative arm of the clock) interact and translocate into the nucleus, where they inhibit CLK:CYC transcriptional promotion, leading to circadian fluctuations in the expression of clock genes [12–16]. Light serves as a fundamental environmental cue for the circadian clock entrainment, which is primarily mediated by the blue light photoreceptor CRY. When activated by light, CRY interacts with TIM, causing its degradation, thus affecting PER stability, thereby resetting the molecular clock [17–20].

As the molecular circadian clock oscillates throughout the day, it provides transcriptional cues to clock-controlled genes that regulate various physiological and metabolic functions without feeding back into the core TFLs [21, 22]. It is reported in mammals, that 5–20% of gene transcripts in any given tissue type are expressed under circadian oscillation [23], so the ablation of a single core clock gene could have a profound effect on an organism’s global transcriptional activity with unknown results.

Recent advancements in genome editing technologies, such as Transcription Activator-Like Effector Nuclease (TALEN) or Clustered Regularly Interspaced Short Palindromic Repeats (CRISPR)/Cas9 have already enabled the generation of silkworm strains with targeted mutations in clock genes. These studies have demonstrated that clock mutations alter the expression profiles of clock genes, clock-controlled genes, physiology and behaviours [24–31]. Building on this foundation, our study is, to our knowledge, the first to demonstrate that knockout (KO) of the circadian *Clock* gene in *B*. *mori* enhances both silk production and pupal weight. By monitoring circadian-regulated behaviours and quantifying variations in important physiological and economic traits between the wild-type and *Clk* null mutants, we showed that *Clk* ablation results in enhanced silk and pupal mass yields. These findings not only contribute to the understanding of clock gene functions beyond circadian rhythm regulation but also highlight the potential for targeted genetic modifications to substantially improve sericultural practices and economic return, while demonstrating the broader applicability of genome editing technology in agricultural biotechnology.

## Material and methods

### Silkworm rearing conditions

The non-diapausing *B*. *mori* Nistari strain was maintained at the Research Centre for Agriculture and Environment, Sericulture Laboratory of Padova, Padova, Italy. Nistari was used for CRISPR/Cas9-mediated *Clk* mutagenesis and represented the wild-type control in the characterisation of the obtained *Clk* mutant strain (*Clk*^*Δ29*^). The TALEN-based *Clk*^*Δ1922*^ mutants and their bivoltine Kosetsu controls [28] were maintained at the Faculty of Textile Science and Technology, Shinshu University, Ueda, Japan. *Clk*^*Δ29*^ and Nistari larvae were reared on 40% artificial diet [32] or fresh mulberry leaves. *Clk*^*Δ1922*^ and Kosetsu silkworms were reared on an artificial diet by Kuwano-hana, JA Zennoh, Gunma, Japan. Silkworms were maintained at 25 ± 1°C and 85% relative humidity (RH). A 12 h light: 12 h darkness (12:12 LD) photoperiod was used with Zeitgeber times (ZTs) 0 and 12 corresponding to light-on and -off, respectively. For experiments performed in constant darkness (DD) conditions, Circadian Time (CT) 0 and CT 12 represented the beginning and end of the subjective day, respectively.

### CRISPR/Cas9-mediated *Clk* mutagenesis

#### Design and generation of *Clk* null mutants

The ~39 kb *Clk* sequence (XM_012693551.1) was retrieved from Ensembl Lepbase (lepbase.org). Sequences for exons 2 and 3 were identified to contain an essential domain for CLK function and selected as the target site for single guide RNA (sgRNA) design. *Clock* gene-specific sgRNAs were identified using the web tool CRISPOR (http://crispor.gi.ucsc.edu/) [33]. Off-target sites were simultaneously monitored for and sgRNAs were selected to avoid genomic exons and coding regions. The sgRNAs (sgRNA 1 and 2; S1 Table) were obtained from Synthego Corporation (Redwood City, California, USA).

The sgRNAs efficacies were confirmed by an *in vitro* digestion; 180 pmol of each sgRNA was incubated with 20 pmol of Cas9 enzyme (EnGen Cas9 NLS, NEB, Massachusetts, United States) at 25°C for 25 min to form RNP complexes. The RNPs were tested against a PCR ~1400 bp target *Clk* amplicon, obtained as follows: genomic DNA (gDNA) was extracted from Nistari larvae using the GeneJet DNA extraction kit (Thermo Scientific™), following manufacturer’s instructions. A ~1400 bp target *Clk* fragment was obtained via PCR using Phusion Green Hot Start II High-Fidelity PCR Master Mix (Thermo Scientific™), 20ng gDNA, 1μM BmClk_For and BmClk_Rev primers (S2 Table). The amplification reaction was performed as: 98°C–2 min, (98°C–20 s, 62°C–30 s, 72°C–30 s) × 35 cycles, 72°C 2–min on a Thermal Cycler Veriti™ (Thermo Scientific™). Amplicons were purified using the GeneJet PCR purification kit (Thermo Scientific™) and eluted in nuclease-free H_2_O.

Two concentrations of RNP complexes (0.5 and 1.0 mM) were incubated with 750 ng of ~1400 bp *Clk* fragment at 37°C for 1 h. The reactions were stopped by incubating at 65°C for 10 min and adding 1 μl of Proteinase K 20 mg/mL (Thermo Scientific™). Samples were run on a 1.2% agarose gel.

#### Establishment of a stable *Clk* null mutant line

To complete germline transformation, Nistari silkmoths were mated upon adult eclosion and eggs were collected within 2 h of laying. Each preblastodermal egg was injected with ~2 nl of RNP solution containing gRNAs 1 and 2 and Cas9 injection buffer (10 mM Tris-HCl, 0.1 mM EGTA, pH 6.8) using an IM-300 microinjector (Narishige, Tokyo, Japan). Injected eggs were incubated at 25°C (± 1°C) with 85% RH until hatching. G_0_ individuals were reared on artificial diet and surviving moths were individually outbred with wild-type silkmoths to generate G_1_ lines.

To identify putative mutations following microinjection, the G_1_ egg batches were incubated for 3 days. Ten eggs were then pooled from each batch to extract gDNA which was used as a template for screening as described in [34], using BmClk_For and BmClk_Rev primers (S2 Table), and Sanger sequencing. A positively identified G_1_ egg batch bearing a large deletion in exons 2 and 3 of *Clk* was identified and the batch was reared until the beginning of the 5^th^ instar and screened by PCR to identify individual larvae bearing the modification. The mutation was outbred for four generations to minimise the possibility of off-target effects before stabilizing a homozygous/hemizygous mutant line.

The *Clk* mutation was also characterised from cDNA by cloning and Sanger sequencing. To generate cDNA, 8 adult brains were pooled and homogenised for both wild-type and hemizygous mutant female moths. Total RNA was extracted using the TRIzol (Thermo Scientific™) method, following the manufacturer’s protocol. Reverse transcription PCR was performed using the Maxima H Minus First Strand cDNA Synthesis Kit (Thermo Scientific™), following the manufacturer’s instructions. The resulting cDNA was used as the template for PCR, using Phusion Master Mix and 1μM BmClk_For and BmClk_Rev primers, as previously described. Amplicons were then ligated into CloneJET PCR cloning vectors (Thermo Scientific™) and transformed into chemically competent Top10 cells by the heat shock method. Plasmids were purified from transformed colonies using the GeneJET Plasmid Miniprep Kit (Thermo Scientific™) following the manufacturer’s instructions, and Sanger sequenced using the pJET1-2F primer (S2 Table) at Eurofins Genomics (Konstanz, Germany). The sequences were aligned in CLC Sequence Viewer 8. Due to a 29 bp deletion in the *Clk* coding sequence, the line was named *Clk* ^*Δ29*^.

### Characterisation of circadian clock gene expression in *Clk*^*Δ29*^

Fifth day Nistari and *Clk* ^*Δ29*^ pupae were removed from their cocoons and maintained in 12:12 LD for at least 6 days. Once emerged, adults were singly transferred into small cups and kept for an additional 12–24 h before collection. Moth heads were deprived of antennae at 8 different ZTs (0, 3, 6, 9, 12, 15, 18, and 21) and stored at– 20°C in RNAlater™ (Thermo Scientific™). For each genotype and time point, 3 replicates with 5–6 heads each, were collected. Total RNA was extracted using TRIzol and a Direct-zol RNA Miniprep Kit (Zymo Research), following the manufacturer’s instructions. Reverse transcription PCR was performed using the Maxima H Minus First Strand cDNA Synthesis Kit (Thermo Scientific™), following the manufacturer’s instructions. Each quantitative PCR (qPCR) reaction was performed in triplicate in a 10 μL reaction volume, containing 200 nM of gene-specific primers (S2 Table) and 20 ng cDNA, using the GoTaq® qPCR Master Mix (Promega) and a CFX96 Real-Time PCR Detection System (Bio-Rad). The amplification conditions were: 95°C 2 min, (15 sec at 95°C, 1 min at 60°C) x 40 cycles. The standard housekeeping genes *rp49* and *Actin 3* were used to normalise the qPCR data [27, 35]. Fold changes were calculated by the 2^-ΔΔ*CT*^ method, using the mean ΔCT of the wild-type strain at ZT3 as a calibrator sample.

### Assessing adult eclosion and egg hatching behaviours in *Clk*^*Δ29*^

To evaluate adult eclosion, fifth-day Nistari and *Clk*^*Δ29*^ pupae were removed from their cocoons and maintained in 12:12 LD for at least 5–6 days, as above. To examine eclosion in constant darkness, pupae were moved to DD at Circadian Time (CT) 0, approximately 24–36 h before eclosion. From 4 to 5 biological replicates (30–70 pupae each) per genotype per condition were analysed.

To assess egg hatching behaviour, adult mating and female egg laying were performed in 12: 12 LD conditions and eggs were collected within 3 h of laying. For each genotype, 4 to 6 biological replicates (200–400 eggs each) derived from 3–4 pooled egg batches were prepared and maintained in 12:12 LD, for at least 6–7 days, when hatching occurred. For DD, egg biological replicates were moved to DD at CT 0 after 5 days.

Adult eclosion and egg hatching were monitored for 2 days. In DD, both eclosion and hatching were monitored under a red LED light (BestSun). Eclosed moths and hatched larvae were counted and for each replicate the percentage of eclosed/hatched individuals was calculated every 2 h in a 24 h profile.

### Assessing larval developmental timing and weights of 5^th^ instar larvae in *Clk*^*Δ29*^ and *Clk*^*Δ1922*^ mutants

Newly hatched *Clk*^*Δ29*^ and *Clk*^*Δ1922*^ larvae and their respective wild-type controls were reared on artificial diet in 12:12 LD and monitored until the beginning of the wandering stage, considered as the moment when about 20% of larvae began spinning the cocoons. At least 100 larvae per genotype were analysed. At the beginning of the 5^th^ instar, from 35 to 43 larvae per genotype were divided by sex and weighted daily till the end the stage.

### Assessing silk productivity and pupal weight in *Clk*^*Δ29*^ and *Clk*^*Δ1922*^ mutants

To assess potential variations in silk productivity and pupal weight, silkworms were reared on artificial diet or fresh mulberry leaves in 12:12 LD with 85% RH. The larvae were transferred to spinning nets to pupate at the wandering stage (late 5th instar). After 4 days, between 80 to 100 pupae per genotype per condition were cut from their cocoons. Weights of total cocoon, silk shell, and pupa were recorded for each individual. Silk productivity was calculated as a ratio between silk shell weight and cocoon total weight, expressed as a percentage. Percentage differences in mean weights for females and males were calculated as [(mean *Clk*^*-*^ mutant female—mean wild-type female) / mean wild-type female]*100.

### Statistical analyses

Data analyses were completed using GraphPad Prism 10.2.2; qPCR, adult eclosion and egg hatching data showed a normal distribution in Shapiro-Wilk and Kolmogorov-Smirnov tests and were analysed by one-way ANOVA and Tukey’s multiple comparison test (with correction for multiple comparisons). Circadian rhythmicity was evaluated using the JTK_CYCLE algorithm version 3.1 [36]. The expression levels of the core clock genes in *Clk*^*Δ29*^ mutants and wild-type controls were analysed with two-way ANOVA followed by Šídák’s multiple comparisons test (with correction for multiple comparisons). Larval weight increments during the 5^th^ instar among sex in *Clk* mutant and wild-type silkworms were compared using repeated measures ANOVA or mixed-effects model, both followed by Šídák’s multiple comparisons test (corrected for multiple comparisons). Silk productivity and pupal weights were compared among sex in wild-type and *Clk*^*-*^ mutants by one-way ANOVA and Šídák’s multiple comparisons test (corrected for multiple comparisons).

## Results and discussion

### CRISPR/Cas9-mediated mutagenesis of *Clock*

**Fig 1:**
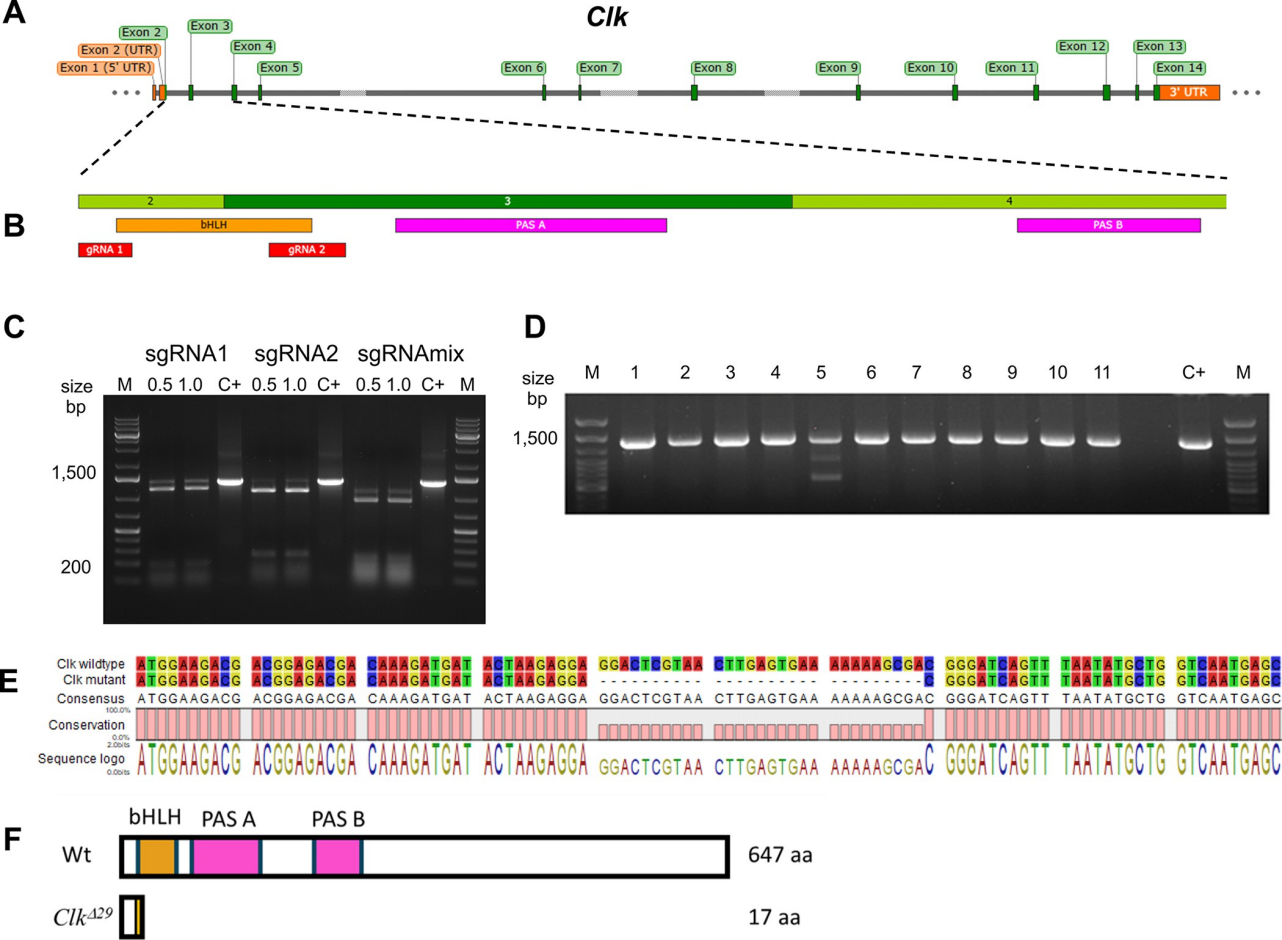
Generation and characterisation of the CRISPR/Cas9-mediated *Clk* null *Bombyx mori*. (A) Schematic representation of the *B*. *mori Clk* genomic region spanning ~39 kb. The 5’ and 3’ UTRs map in exons 1, 2 and 14, respectively. (B) *Clk* CDS spanning 1941 bp from exons 2 to 14; bHLH and PAS functional domains are shown in orange and pink, respectively; sgRNAs localising to exons 2 and 3 are represented in red. (C) Semiquantitative agarose gel showing *in vitro* efficacy of sgRNA 1 and sgRNA2, tested individually and combined. The sgRNA-Cas9 RNP complexes were incubated with 750 ng of ~1400 bp *Clk* amplicons. The two sgRNAs successfully localised and digested the DNA template, with little or no differences between the two tested concentrations (0.5 and 1.0 mM). The combination of the two sgRNAs (sgRNA mix) appeared more effective in DNA digestion compared to the same sgRNAs, individually evaluated (sgRNA1 and sgRNA2). C+ indicates the control (undigested ~1400 bp *Clk* amplicon); 0.5 and 1.0 represent the two tested concentrations in mM; M: DNA ladder. (D) PCR screening of G_1_ eggs derived from G_0_ outbred moths. Each band represents a *Clk* amplicon obtained from gDNA of 10 pooled eggs for each successfully laid egg batch. Number 5 has a multiband, indicating a DNA modification in this batch. (E) Sequence alignment of a *Clk* CDS fragment obtained via RT-PCR and standard PCR from wild-type and mutant females. The mutated *Clk* sequence (*Clk*^*Δ29*^*)* carries a 29bp deletion in the CDS spanning the last two bases of exon 2 and early exon 3. Hyphens indicate the deleted bases. (F) Conceptual translation of the CLK proteins from wild-type *Clk* and mutant *Clk*^*Δ29*^ CDSs. Wild-type 647 aa CLK, with the bHLH and PAS functional domains shown in orange and pink, respectively; *Clk*^*Δ29*^: a truncated 17 aa CLK peptide, due to a stop codon produced by the frameshift mutation 12 nucleotides downstream the 29 bp deletion.

The *B*. *mori Clk* gene (Gene ID: XM_012693551.1) spans ~39 kb and maps on the Z sex chromosome with 14 exons, including a 1941 bp coding region (Fig 1A). Using the webtool CRISPOR [33], two sgRNAs were designed targeting exons 2 and 3, which encode the bHLH domain essential for CLK transcriptional activity (Fig 1B and S1 Table). *In vitro*, both sgRNAs effectively digested the *Clk* fragment, with similar profiles at both tested concentrations (0.5 and 1 mM of RNP complexes), suggesting that, at the concentrations assessed, target DNA digestion plateaued within one hour regardless of RNP concentration. The combination of both gRNAs resulted in more efficient DNA digestion compared to individual sgRNAs (Fig 1C). Dual sgRNA strategies have been reported to enhance the generation of KO mutants in several species [37–39], and our data support their use in generating *B*. *mori* KO lines.

Next, approximately 1400 fertilized Nistari eggs were microinjected using the dual gRNAs approach. Thirty-one G_0_ individuals survived to adulthood and 23 successfully mated, resulting in fertile G_1_ egg batches. An egg batch containing a *Clk* modification (Fig 1D) was outbred with wild-type silkmoths for four generations as in [34], before establishing the stable *Clk*-modified mutant line. Characterisation of the *Clk* modification by Sanger sequencing from gDNA and cDNA identified a ~700 bp deletion in the *Clk* gene corresponding to a 29 bp deletion in exons 2 and 3 of the *Clk* coding region (*Clk*^*Δ29*^; Fig 1E). The 29 bp deletion resulted in a frameshift downstream of the mutation site, resulting in an early stop codon in the bHLH domain coding region, generating a putative 17 amino acid (aa) CLK peptide fragment lacking all CLK functional domains (Fig 1F). Using a dual sgRNAs-CRISPR/Cas9 genome editing approach, we obtained a mutagenesis efficiency of 3.2% in surviving larvae.

### Characterisation of *Clk*^*Δ29*^ mutants

#### *Clk*^*Δ29*^ exhibits disrupted temporal transcription profiles of core circadian clock genes

**Fig 2:**
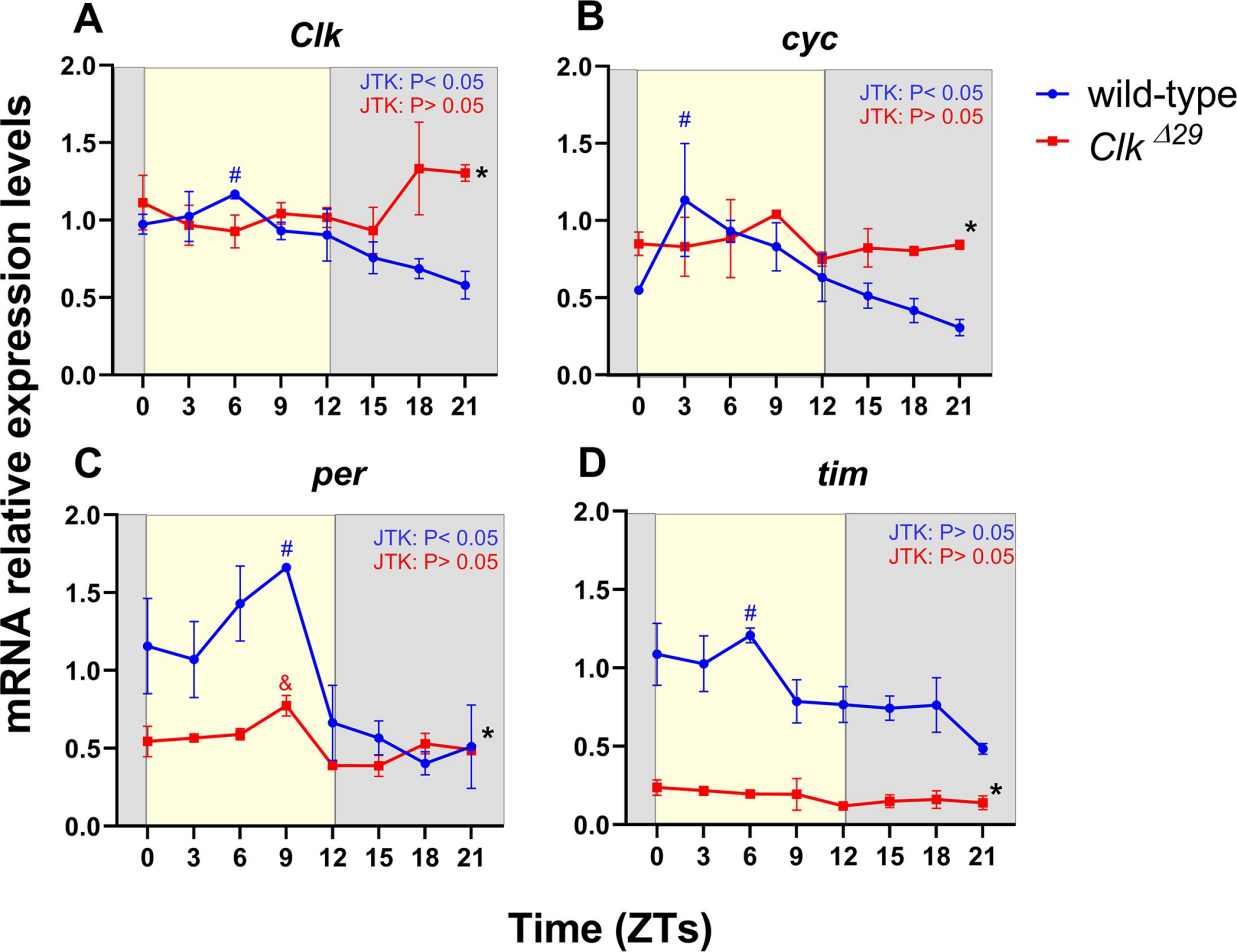
Daily expression of core clock genes in wild-type and *Clk*^*Δ*^^*29*^ heads under 12:12 LD conditions. Relative mRNA expression levels [mean ± standard error of the mean (SEM)] of (A) *Clk*, (B) *cyc*, (C) *per*, and (D) *tim* in heads of wild-type (blue circles) and *Clk*^*Δ29*^ mutant (red squares) moths at eight different time points (ZTs 0, 3, 6, 9, 12, 15, 18, 21). Sampling at ZT 0 and 12 occurred a few minutes before light-on and -off, respectively. For each clock gene, *actin3* and *rp49* were used as internal controls, and the mRNA relative levels were normalised to the mean mRNA levels in wild-type controls at ZT3, using the ΔΔCt method. Three biological replicates per time point, each with 4 to 6 heads without antennae, were analysed for both genotypes. Yellow and grey areas respectively indicate light and dark phases in 12:12 LD conditions. Significant differences in daily mRNA relative levels within the same genotype (blue: wild-type; red: *Clk*^*Δ29*^ mutant) were determined with one-way ANOVA followed by Tukey’s multiple comparisons test and JTK_CYCLE algorithm. Significant pairwise comparisons (P<0.05) in the Tukey’s *post hoc* test are indicated as follows: A) #: ZT 6 *vs* ZT 21; B) #: ZT 3 *vs* ZT 21; C) #: ZT 9 *vs* ZTs 15, 18, and 21; &: ZT 9 *vs* ZTs 12, 15, and 21; D) #: ZT 6 *vs* ZT 21. In each panel, JTK: P < 0.05 and JTK: P > 0.05 indicate respectively rhythmic and arrhythmic mRNA expressions determined with JTK_CYCLE algorithm. The black asterisk (*) indicates P< 0.05, significant differences in the relative expression levels between wild-type and *Clk*^*Δ29*^ mutant heads determined by two-way ANOVA, genotype effect.

To evaluate the effect of the *Clk*^*Δ29*^ mutation on the *B*. *mori* molecular circadian clock, the daily expression patterns of *Clk*, *cyc*, *per* and *tim* core clock genes were examined under 12: 12 LD via qPCR, in wild-type and *Clk*^*Δ29*^ adult heads deprived of their antennae, which are known to contain an autonomous circadian clock in Lepidoptera [10] (Fig 2).

In wild-type heads, *Clk* and *cyc* exhibited significant rhythmic expression (one-way ANOVA: P < 0.05; JTK_CYCLE: P < 0.01), with significantly higher mRNA levels during the day compared to the late night (Tukey’s *post hoc* test: ZTs 6 and 3 *vs* ZT 21: P<0.05 for *Clk* and *cyc*, respectively) (Fig 2A and 2B). Initial studies using Northern blot detected *Clk* and *cyc* expressions with weak/absent rhythmicity in *B*. *mori* adult heads [40]. However, recent qPCR analyses in larval heads and pupal ovaries identified rhythmic expression for both genes, with higher levels during the light phase under 12:12 LD conditions [26, 27]. Though we cannot exclude that some strain-specific differences exist, these findings suggest that *Clk* and *cyc* exhibit rhythmic expression across various tissues and developmental stages in *B*. *mori*.

Under 12:12 LD conditions, wild-type heads showed significant daily cycling *per* expression, with higher mRNA levels during daytime and lower levels at night (one-way ANOVA: P < 0.01; JTK_CYCLE: P < 0.01; Tukey’s *post hoc* test: ZT9 vs ZTs 15, 18, and 21: P < 0.05) (Fig 2C). In contrast, *tim* mRNA exhibited a weak/absent daily oscillation that was significant by one-way ANOVA (P < 0.05) but not by JTK_CYCLE [P = 0.06, not significant (ns)] (Fig 2D). Previous studies on adult heads of a different *B*. *mori* strain (N4) detected significant cycling in both *per* and *tim* mRNAs, with peaks around ZT 12 [41]. However, in embryos belonging to the Nistari strain *per* and *tim* mRNAs were shown to peak during daytime and nighttime, respectively [25]. Differences in *per* and *tim* expression profiles across studies may be attributed to variations in strains, tissues, and developmental stages, as previously suggested [24–27, 41–43].

In *Clk*^*Δ29*^ mutants under 12:12 LD conditions, the temporal expression profiles of all four core clock genes were altered compared to wild-type controls (Fig 2). *Clk* and *cyc* mRNA levels showed significant increases during the late night in *Clk*^*Δ29*^ mutants, while no significant differences were observed during the day (two-way ANOVA: genotype effect: P < 0.05; *Clk*: Šídák’s *post hoc* test: wild-type *vs Clk*^*Δ29*^ at ZTs 18 and 21: P < 0.01; *cyc*: Šídák’s *post hoc* test: wild-type vs *Clk*^*Δ29*^ at ZT 21: P < 0.05) (Fig 2A and 2B). This likely caused the loss of rhythmic expression for both genes in *Clk*^*Δ29*^ heads (*Clk* and *cyc*: P > 0.05, ns, in both one-way ANOVA and JTK_CYCLE), suggesting that the absence of functional CLK affects the rhythmic expression of both *Clk* and *cyc*.

The mechanisms regulating *cyc* daily transcription in Lepidoptera remain unclear. It is believed that *Clk* mRNA rhythmicity is modulated by VRILLE (VRI) and PDP1, which respectively inhibit and promote *Clk* transcription, similar to *Drosophila* [44–46]. The lack of *Clk* mRNA daily cycling in *Clk*^*Δ29*^ mutants may be partly explained by aberrant VRI and PDP1 production due to the absence of functional CLK.

In *Clk*^*Δ29*^ mutants, *per* and *tim* expression levels were significantly lower compared to controls (two-way ANOVA: genotype effect: P< 0.001) (Fig 2C and 2D). The decrease in *per* mRNA was particularly evident during the light phase (Šídák’s post hoc test: *per* in wild-type *vs Clk*^*Δ29*^ at ZTs 0 to 9: P < 0.05), resulting in dampened daily oscillation (one-way ANOVA: P < 0.01; JTK_CYCLE: P = 0.41, ns) (Fig 2C). *tim* mRNA levels were constitutively low throughout the day in *Clk*^*Δ29*^ mutants (Šídák’s post hoc test: *tim* in wild-type *vs Clk*^*Δ29*^ at all ZTs: P < 0.05) (Fig 2D). These findings are consistent with observations in *Drosophila* and the monarch butterfly, where *Clk* mutations resulted in low *per* and *tim* mRNA expression [14, 47], underscoring the essential role of CLK in the CLK:CYC activator complex within the *B*. *mori* circadian clock.

Recently, three other *Clk* mutant alleles were produced in *B*. *mori*, *Clk*^*Δ1922*^ and *Clk*^*Δ17851*^, both with early stop codons in the bHLH domain [28], and *Clk*^*Δ5*^, which lacks most of the PAS-B domain due to a 5 bp deletion in exon 7 [27]. The impact of these mutations on core clock gene expressions was not assessed in *Clk*^*Δ1922*^ and *Clk*^*Δ17851*^ strains. However, analyses in fifth instar *Clk*^*Δ5*^ silkworms showed dampened *Clk* mRNA cycling and strong downregulation of *tim* expression, similar to our *Clk*^*Δ29*^ results [27]. Notably, *Clk*^*Δ5*^ mutants also exhibited a general upregulation of *cyc* mRNA throughout the day and increased *per* transcription during the dark phase, which differs from our findings with *Clk*^*Δ29*^ [27]. These differences may be due to the distinct genetic backgrounds of the Nistari polyvoltine (*Clk*^*Δ29*^) and p50T (Daizo) bivoltine (*Clk*^*Δ5*^) strains used for CRISPR/Cas9 mutagenesis [27]. Additionally, *Clk*^*Δ5*^ may produce a PAS-B-truncated CLK protein with aberrant transcriptional activity, whereas *Clk*^*Δ29*^ likely produces a non-functional 17 aa CLK peptide fragment. Future studies using CLK-specific antibodies could clarify these differences. Interestingly, a *Clk* mutant allele in the monarch butterfly with an early stop codon after the bHLH domain also resulted in low *per* and *tim* expression, similar to our findings [47].

**Adult eclosion and egg hatching rhythms are disrupted in *Clk***^*Δ****29***^. In *B*. *mori*, wild-type individuals show a daily rhythmicity in adult eclosion and egg hatching behaviours, under 12:12 LD and DD conditions [48–50]. On the other hand, in clock mutants these phenotypes were shown to be rhythmic under 12:12 LD regimes and arrhythmic under DD conditions [24, 25], indicating that in *B*. *mori* a functional clock is essential in driving rhythmicity in the absence of synchronising environmental cue, such as the alternating light dark cycle. To assess the role of CLK in these circadian behaviours, we analysed eclosion and egg hatching rhythms in wild-type and *Clk*^*Δ29*^ strains, in both 12:12 LD and DD regimes.

**Fig 3:**
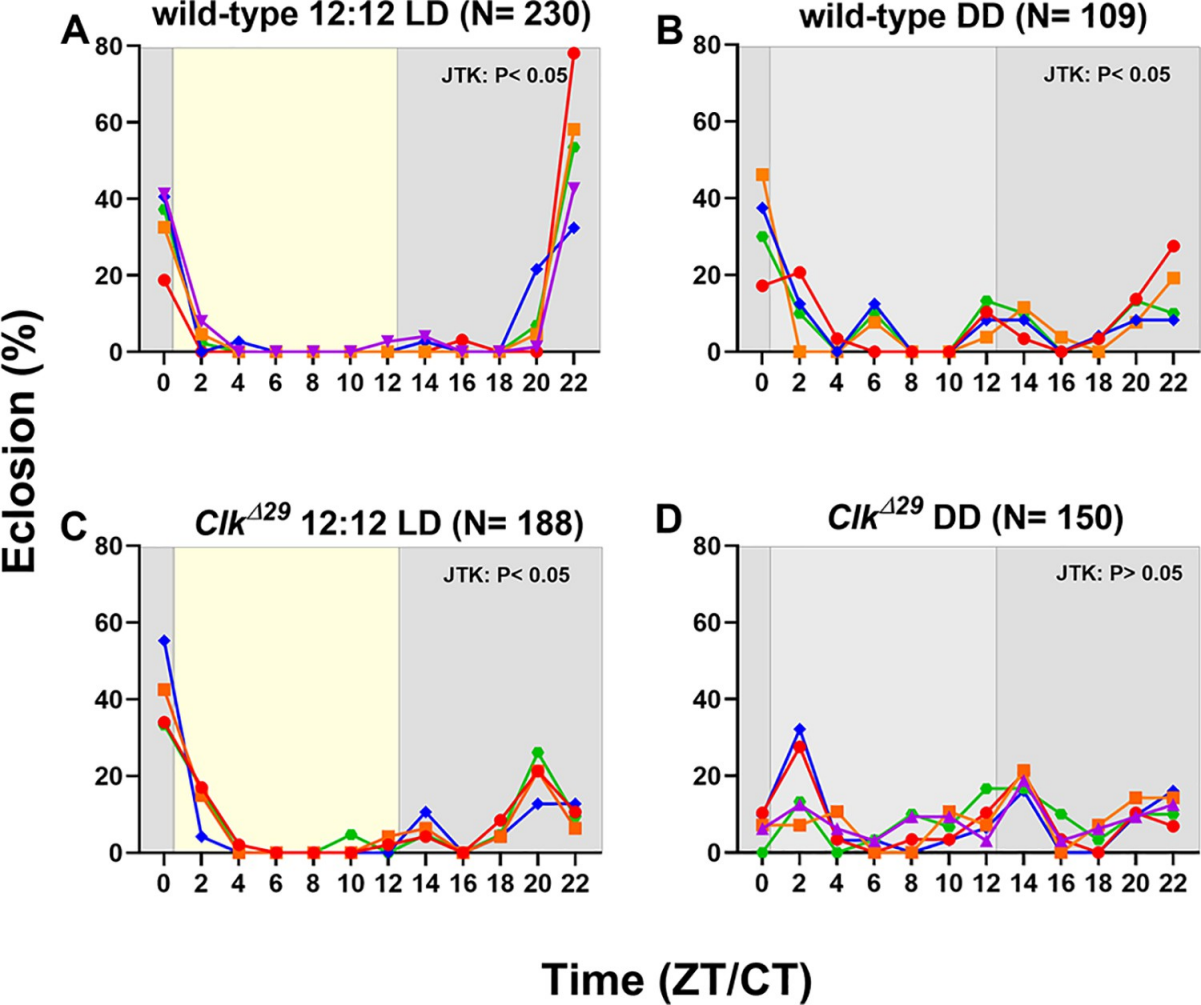
Adult eclosion rhythm is disrupted in *Clk*^*Δ29*^ under DD conditions. Percentages (%) of eclosion in wild-type controls under (A) 12:12 LD and (B) DD, and in *Clk*^*Δ29*^ mutants under (C) 12:12 LD and (D) DD conditions. Eclosion is reported every two hours at 12 different time points (ZT/CT 0, 2, 4, 6, 8, 10, 12, 14, 16 18, 20, 22). Eclosion at ZTs 0 and 12 was evaluated immediately before light-on and -off, respectively. In each panel, eclosion percentages of each single replicate are reported in different colours (blue, red, orange, green, and purple). From 30 to 70 individuals per replicate were analysed. N between parentheses indicates the total number of analysed individuals per genotype per condition. In 12:12 LD conditions, yellow and grey areas indicate light and dark phases, respectively. Under DD regimes, subjective day and night are shown by light and dark grey areas, respectively. JTK: P < 0.05 and JTK: P > 0.05 indicate respectively rhythmic and arrhythmic eclosion determined with JTK_CYCLE algorithm.

Under 12:12 LD conditions, wild-type moths showed significant rhythmic eclosion (P < 0.0001, in both one-way ANOVA and JTK_CYCLE), predominantly occurring at the end of the night with a peak at ZT 22 (Fig 3A). In DD conditions, wild-type eclosion maintained a 24 h rhythmicity (one-way ANOVA: P < 0.0001; JTK_CYCLE: P < 0.01), with the peak of activity occurring at CT 0, corresponding to the onset of the subjective day (Fig 3B). *Clk*^*Δ29*^ mutant moths displayed rhythmic eclosion under 12:12 LD conditions (one-way ANOVA: P < 0.0001; JTK_CYCLE: P < 0.05) (Fig 3C). However, compared to wild-type, *Clk*^*Δ29*^ mutants had a 2 h delayed peak activity at ZT 24/0. In DD conditions, *Clk*^*Δ29*^ mutants showed significant variations in eclosion throughout the day (one-way ANOVA: P < 0.0001), but the eclosion pattern was arrhythmic according to the JTK_CYCLE algorithm (P = 1, ns) (Fig 3D).

**Fig 4:**
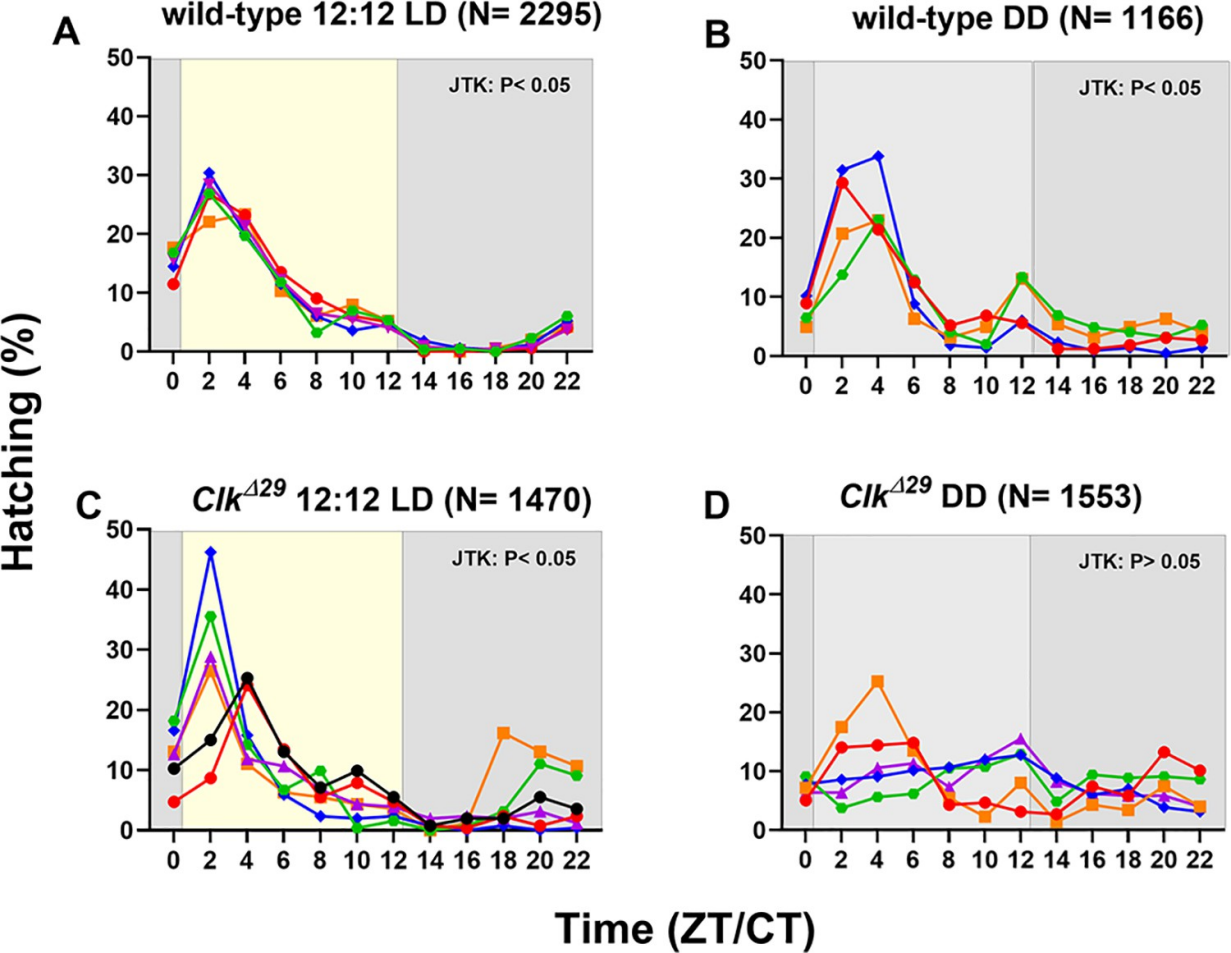
Rhythmic egg hatching is disrupted in *Clk*^*Δ29*^ under DD conditions. Percentages (%) of egg hatching in wild-type controls under (A) 12:12 LD and (B) DD, and in *Clk*^*Δ29*^ mutants under (C) 12:12 LD and (D) DD conditions. Hachting is reported every two hours at 12 different time points (ZT/CT 0, 2, 4, 6, 8, 10, 12, 14, 16 18, 20, 22). Egg hatching at ZTs 0 and 12 was evaluated immediately before light-on and -off, respectively. In each panel, hatching %s of each single replicate are reported in different colours (blue, red, orange, green, purple, and black). From ~700 to 200 eggs per replicate were analysed. N between parentheses indicates the total number of analysed eggs per genotype per condition. In 12:12 LD conditions, yellow and grey areas indicate light and dark phases, respectively. Under DD regimes, subjective day and night are shown by light and dark grey areas, respectively. JTK: P < 0.05 and JTK: P > 0.05 indicate respectively rhythmic and arrhythmic hatching determined with JTK_CYCLE algorithm.

When we examined egg hatching rhythms, the wild-type strain showed a significantly rhythmic egg hatching activity, under both 12:12 LD and DD conditions (for both LD and DD regimes: one-way ANOVA: P < 0.0001; JTK_CYCLE: P < < 0.00001). The hatching peak occurred at ZT 2 in 12:12 LD and between CTs 2 and 4 in DD (Fig 4A and 4B). In *Clk*^*Δ29*^ mutants, egg hatching under 12:12 LD conditions remained rhythmic (one-way ANOVA: P < 0.0001; JTK_CYCLE: P < < 0.00001) with a peak at ZT 2 (Fig 4C). However, under DD conditions, *Clk*^*Δ29*^ mutants exhibited arrhythmic hatching, occurring erratically throughout the 24 h period (one-way ANOVA: P = 0.095, ns; JTK_CYCLE: P = 0.183, ns) (Fig 4D).

These data indicate that a functional CLK is essential for *B*. *mori* circadian behaviours, aligning with findings in the monarch butterfly where a *Clk* KO affected the rhythmicity of adult eclosion, when evaluated under DD regimes [47]. This highlights the central role of CLK in controlling circadian behaviour in Lepidoptera.

### *Clk*^*-*^ null mutants exhibit prolonged larval development, increases in pupal weight and silk productivity

Circadian clock genes have been shown to modulate various physiological phenomena crucial for sericultural production. In *B mori*, bivoltine strains exhibit a facultative transgenerational egg diapause, which is influenced by environmental factors such as temperature and photoperiod experienced by the mother during embryonic and larval development [51]. Mutations in clock genes affect egg diapause induced by temperature (*per*, *tim*, *Clk*, *cyc* and *cry2)* and photoperiod (*per*, *tim*, *Clk*, *cyc*, and *cry1*) [26–28, 31, 42], indicating a circadian clock role in these behaviours. Additionally, the circadian clock has been demonstrated to impact *B*. *mori* developmental timings. For instance, a *cry1* KO line exhibited delayed developmental time in both late larval instars and pupae [30]. In contrast, the knockdown of *per* was shown to accelerate the timing of embryonic development, the final larval moulting period, and pupal metamorphosis [52].

To determine if *Clk* regulates physiological parameters relevant to both sericulture and the generation of silkworm-derived products, we evaluated several phenotypes related to development and productivity in *Clk*^*Δ29*^ mutants and wild-type Nistari, maintained on artificial diet under 12:12 LD conditions. Additionally, the same traits were analysed under comparable rearing conditions in *Clk*^*Δ1922*^, a second *B*. *mori Clk* mutant line, characterised by an early stop codon in the *Clk* coding region, similar to *Clk*^*Δ29*^ [28]. The *Clk*^*Δ1922*^ mutant was obtained using the TALEN genome editing in the Kosetsu bivoltine strain [28].

**Fig 5:**
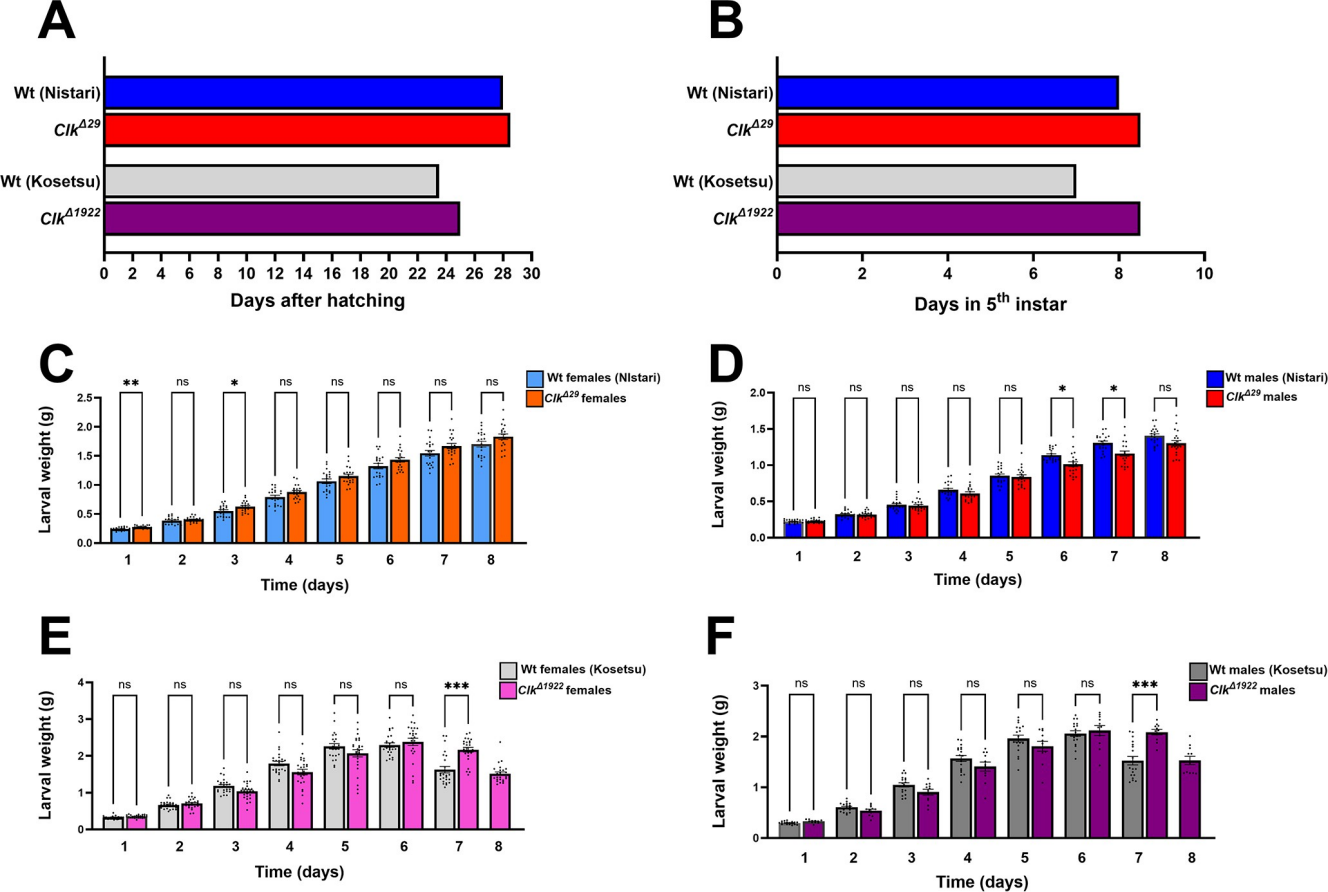
*Clk* modulates larval development. Developmental timing (A) from egg hatching to beginning of pupation and (B) of the last (5^th^) larval stage in *Clk*^*Δ29*^ (red) and *Clk*^*Δ1922*^ (purple) mutants and in their respective Nistari (blue) and Kosetsu (gray) wild-type controls. At least 100 larvae were monitored for each genotype. The beginning of the wandering stage was considered the moment when about 20% of larvae began spinning the cocoons. Larval weight increments during the 5^th^ instar in (C) *Clk*^*Δ29*^ (orange) and Nistari (light blue) females, (D) *Clk*^*Δ29*^ (red) and Nistari (blue) males, (E) *Clk*^*Δ1922*^ (pink) and Kosetsu (light gray) females, (F) *Clk*^*Δ1922*^ (purple) and Kosetsu (dark gray) males. From 25 to 11 larvae per sex per genotype were analysed. Repeated measures one-way ANOVA (C, D) and mixed-effects analyses (E, F): P = <0.0001; Šídák’s *post hoc* test: P < 0.05 (*), P < 0.01 (**), P < 0.001 (***), ns, not significant.

We first assessed whether *Clk* is implicated in *B*. *mori* developmental timing. When we analysed the daily rhythmicity of adult eclosion (see above), we did not observe any difference in the duration of the pupal stage between *Clk*^*Δ29*^ and wild-type Nistari strains (lasting about 11 days in both strains). Therefore, we focused on larval development, from egg hatching to the beginning of wandering stage (corresponding to the moment when approximately 20% of larvae in the batch begin spinning cocoons) (Fig 5). Our analyses showed that both *Clk*^*Δ29*^ and *Clk*^*Δ1922*^ strains were characterised by a longer developmental time compared to their respective controls (Fig 5A). For both mutants, the delay occurred mainly during the 5^th^ larval stage, with *Clk*^*Δ29*^ and *Clk*^*Δ1922*^ silkworms respectively showing a delay of about 12–24 and 36 h compared to their appropriate wild-type controls (Fig 5B). This variable degree of developmental delay observed in the two *Clk*^*-*^ mutants likely reflects differences in the genetic background of the two distinct wild-type recipient strains used for their generation.

To explore whether the prolonged developmental time was associated with differences in silkworm growth, we compared the weight of *Clk* mutant and wild-type 5^th^ instar larvae (Fig 5C–5F). During the eight days of larval development, *Clk*^*Δ29*^ mutants did not exhibit consistent weight differences compared to controls. *Clk*^*Δ29*^ mutant females showed slight weight increases, generally non-significant apart from those recorded on days 1 and 3 of the 5^th^ instar (Fig 5C). *Clk*^*Δ29*^ mutant males displayed no relevant weight changes compared to age-matched controls, except for a significant decrease on days 6 and 7 of larval development (Fig 5D). Regarding *Clk*^*Δ1922*^ mutants, neither females nor males showed substantial differences in body weight compared to controls during the first six days of the last larval stage (Fig 5E and 5F). Significant variations were detected only on day 7, with *Clk*^*Δ1922*^ females and males displaying higher weights than controls (P = 0.0005 for both females and males, Šídák’s *post hoc* test). However, these differences were likely due to the 36 h-developmental delay of *Clk*^*Δ1922*^ mutants compared to their controls. For the mutants, day 7 still represented a phase of active feeding and growth, while for the controls, it corresponded to the final day of the 5^th^ larval stage. At this moment, *B*. *mori* larvae typically exhibit weight loss as they stop feeding and undergo gut clearing in preparation for metamorphosis [53]. Taken together these data indicate that in both *Clk*^*-*^ mutants, the absence of a functional CLK induces a prolonged larval phase during the last instar, when silkworms actively feed. However, this prolonged feeding time does not lead to dramatic variations in weight. Future studies will clarify the molecular pathways underlying this phenotype, determining whether they influence the regulation of molting hormone levels, including ecdysteroids and juvenile hormones, as previously shown in silkworms carrying a null mutation at the level of the key circadian clock gene *cry1* [30].

**Fig 6:**
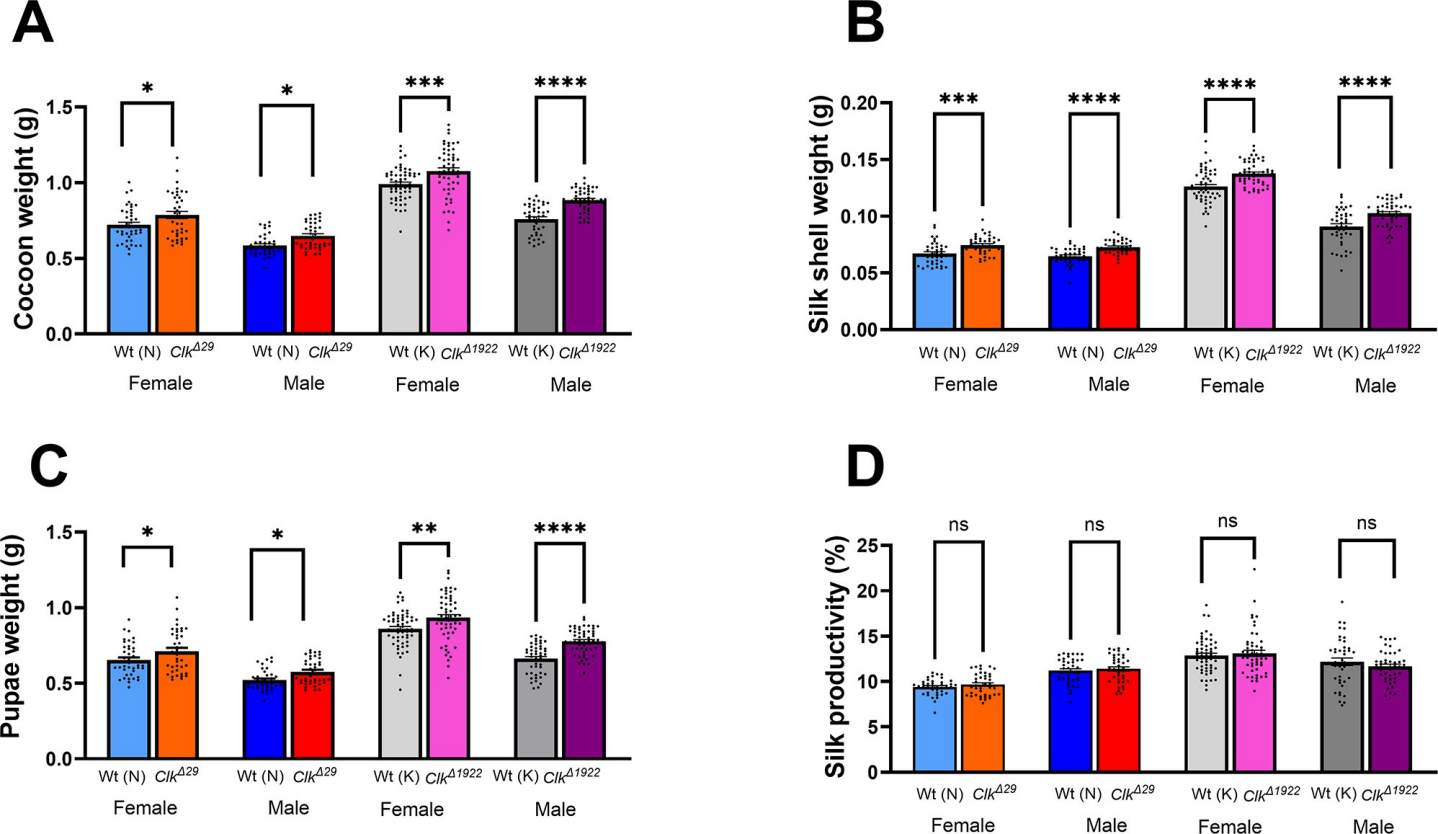
Cocoon, silk shell, pupal weights and silk productivity in *Clk*^*-*^ mutants fed on artificial diet. (A) Whole cocoon weight (mean ± SEM); (B) silk shell weight (mean ± SEM); (C) pupal weight (mean ± SEM); and (D) silk productivity (%) (mean ± SEM) of *Clk*^*Δ29*^ and *Clk*^*Δ1922*^ mutants in comparison to their respective Nistari (N) and Kosetsu (K) controls. *N* = 40–59 per sex per genotype. *Clk*^*-*^ mutants *vs* appropriate controls one-way ANOVA P <0.0001; Šídák’s *post hoc* test (corrected for multiple comparisons): P < 0.05 (*), P < 0.01 (**), P < 0.001 (***), P < 0.0001 (****), ns, not significant.

To understand whether the developmental differences observed during the last larval stage in *Clk*^*-*^ mutants influenced cocoon and pupa formation, we compared the weights of whole cocoons, silk shells and pupae in *Clk*^*-*^ mutants and their wild-type controls (Fig 6). Both *Clk*^*Δ29*^ and *Clk*^*Δ1922*^ mutants showed significantly higher whole cocoon weights (Fig 6A) (One-way ANOVA: P <0.0001; ~ 10% increments in both *Clk*^*Δ29*^ females and males; ~ 9 and 16% increments in *Clk*^*Δ1922*^ females and males, respectively). Significant weight increases were also observed in *Clk*^*Δ29*^ and *Clk*^*Δ1922*^ silk shells (Fig 6B) (One-way ANOVA: P <0.0001; ~ 11% increments in both *Clk*^*Δ29*^ females and males; ~ 9 and 13% increments in *Clk* ^*Δ1922*^ females and males, respectively) and in pupae (Fig 6C) (One-way ANOVA: P <0.0001; ~ 9 and 11% increments in *Clk*^*Δ29*^ females and males, respectively; ~ 9 and 17% increments in *Clk*^*Δ1922*^ females and males, respectively).

**Fig 7:**
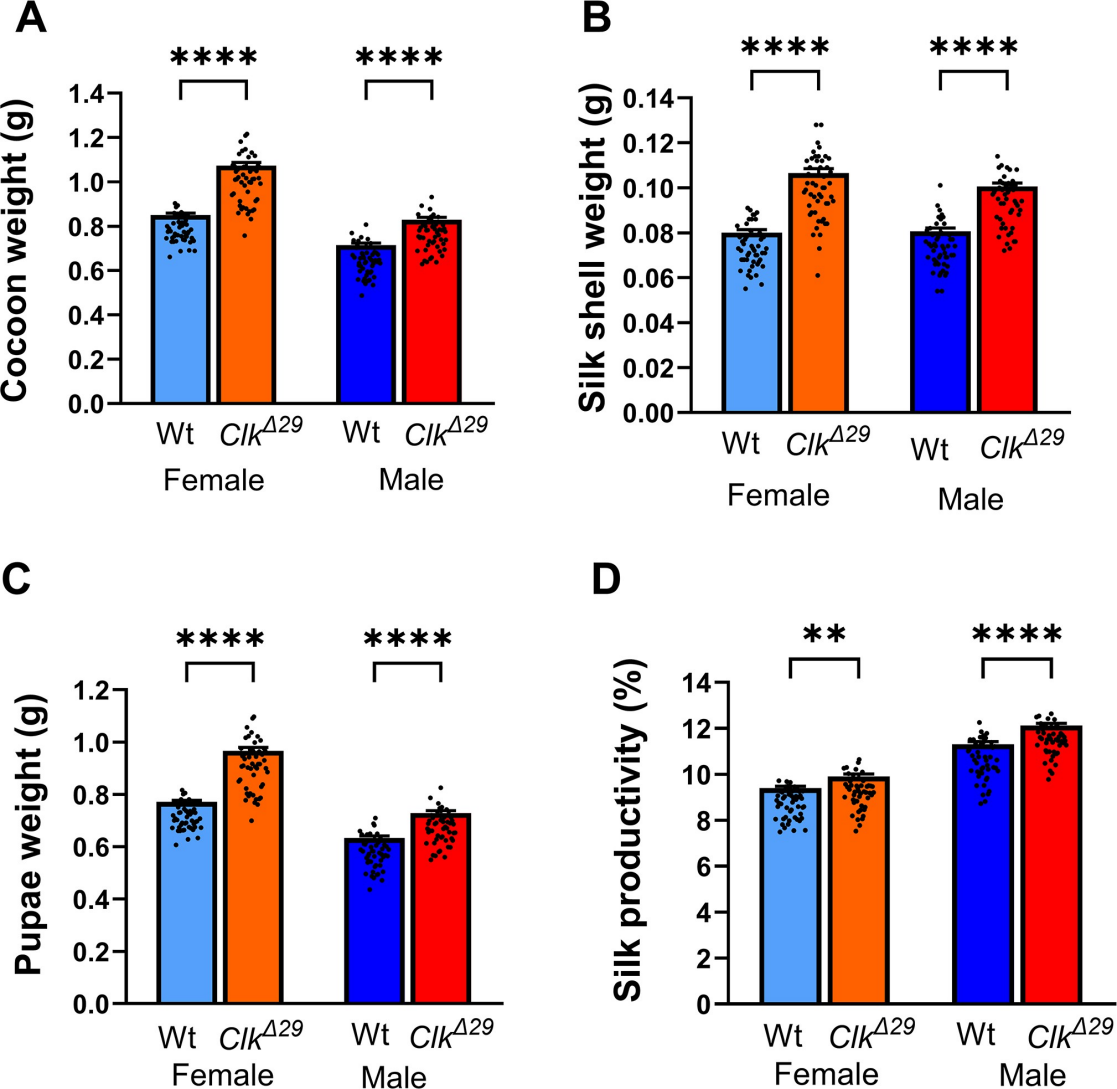
*Clk*^*Δ*^^*29*^ mutants exhibit enhanced silk productivity and pupal weight when fed on mulberry leaves. (A) Whole cocoon weight (mean ± SEM); (B) silk shell weight (mean ± SEM); (C) Pupal weight (mean ± SEM); and (D) silk productivity (%) (mean ± SEM) of *Clk*^*Δ29*^ mutants and wild-type controls. *N* = 50 per sex per genotype; comparisons by one-way ANOVA: P < 0.0001; Šídák’s *post hoc* test: P < 0.01 (**), P < 0.0001 (****). *Clk*^*Δ29*^ females and males are shown with orange and red bars, respectively; control females and males are shown with light blue and blue bars.

Silk productivity, an important parameter in sericulture, is measured as the percentage ratio of shell weight to cocoon weight. When we determined silk productivity in *Clk*^*-*^ mutants and controls reared on artificial diets, we did not detect any significant difference (Fig 6D) (*Clk*^*Δ29*^ and *Clk*^*Δ1922*^ *vs* respective controls: Šídák’s *post hoc* test: P > 0.05, ns in both females and males). However, silk productivity slightly increased in *Clk*^*Δ29*^ (2.9 and 1.8% in females and males, respectively) and in *Clk*^*Δ1922*^ females (1.8%) while males, in contrast, showed a 4.1% decrease. These data prompted us to evaluate productivity parameters in silkworms reared on mulberry leaves, considered an ideal diet for sericulture as it aligns with natural nutritional needs of silkworms, thereby optimising silk yield and quality [54]. We focused on *Clk*^*Δ29*^ mutants and their wild-type controls, which were maintained on mulberry leaves from the beginning of the 5^th^ larval instar. The weights of cocoons, silk shells and pupae were subsequently measured (Fig 7). As expected under these rearing conditions, both mutants and wild-type strains showed substantial increases in all the analysed parameters compared to those observed with the artificial diet. Additionally, when compared to controls, *Clk*^*Δ29*^ mutants exhibited significantly greater overall cocoon weights, increasing by 26.1 and 16.2% in females and males, respectively (Fig 7A). Mean silk shell weights also significantly increased by 32.9 and 24.7% in *Clk*^*Δ29*^ females and males, respectively (Fig 7B) (one-way ANOVA: P < 0.0001). Moreover, *Clk*^*Δ29*^ mutants also showed increased pupal weight (one-way ANOVA: P < 0.0001) (Fig 7C), with females and males exhibiting 25.4% and 15.2% increases, respectively. Importantly, *Clk*^*Δ29*^ mutants exhibited a significant enhancement in silk productivity (5.5% in females and ~7.2% in males) (one-way ANOVA: P < 0.0001; Šídák’s *post hoc* test: females: P = 0.0024; males: P < 0.0001) (Fig 7D).

Taken together, these data suggest that the absence of a functional CLK affects physiological processes involved in the regulation of *B*. *mori* development and growth. In fact, both *Clk*^*-*^ mutants produced in different *B*. *mori* recipient strains and with different genome editing strategies showed similar modifications in the timing of the late larval development and in the weight of pupae and cocoon components. Additionally, the differences in the productivity parameters between *Clk*^*-*^ mutants and controls were more pronounced when silkworms were reared on mulberry leaves, to which they are naturally adapted, supporting optimal digestion and growth [54]. This observation indicates that the mulberry leaf diet amplifies the genetic effects on these traits, making the differences more evident, suggesting an influence of genotype-environment interactions on *B*. *mori* development.

Further analyses are required to identify which genes and pathways are altered in *Clk*^*-*^ mutants and to clarify whether the detected phenotypes are due to a truly circadian clock function rather than to pleiotropic effects of *B*. *mori* CLK. However, in mammals *Clk* is important for circadian regulation of macronutrient absorption in the intestine and *Clk* disruption in mice has been associated with obesity, hyperphagia, and different types of metabolic syndromes [55, 56]. In *Drosophila*, the circadian clock has been demonstrated to control metabolism and energy homeostasis, in a complex regulation which involves the interplay between neuronal and peripheral metabolic/digestive clocks [57]. In *B*. *mori*, the circadian clock has been suggested to regulate essential physiological and metabolic processes for development and growth, as *cry1* has been shown to modulate hormone production, such as ecdysteroids and juvenile hormones, thereby controlling larval and pupal developmental timing [30]. Additionally, a *cry1* knockout was shown to increase glucose metabolism but reduced cell size in *B*. *mori* cells, evidenced by elevated glucose-6-phosphate, fructose-6-phosphate levels, and pyruvic acid levels [29]. Therefore, *Clk* gene ablation may feasibly cause metabolic dysfunction in *Clk*^*-*^ mutants, delaying larval development, extending feeding time, and leading to larger pupae.

Future studies should investigate the impact of the *Clk* mutations on economic parameters across various genetic backgrounds, including commercial monovoltine strains, as well as more complex hybrid and polyhybrid crosses used for silk production. However, the increments in growth and silk productivity parameters we detected in *Clk*^*Δ29*^ mutants when reared on mulberry leaves are in line with those observed in other genome-editing studies to improve key commercial aspects of *B*. *mori*. A CRISPR/Cas9-induced KO of *B*. *mori Ecdysteroid kinase-like-1* (*EcKL1*), a member of the *ecdysteroid kinase-like* family involved in hormone regulation and energy metabolism pathways, induced significant increases of up to 10% in silk yield, without significantly affecting pupal size [6]. Additionally, a CRISPR/Cas9-mediated knockdown (KD) of *let-7*, a miRNA regulating developmental timing with conserved functions among *Bilateria*, induced significant increments of up to 50% in cocoon and pupal size and up to 10% in silk productivity [58]. However, *let7*-KD pupae died within the cocoon at a pupa-moth intermediate state and were not able to reach an adult stage giving rise to a stable *let7*-KD *B*. *mori* strain [58]. Nevertheless, taken together, these studies emphasise the profound impact of genome-editing approaches for targeted gene disruptions affecting economically important traits in *B*. *mori* and highlight the potential and opportunities that remain to be explored in the domestic silkworm’s genome.

### Conclusions

Using a CRISPR/Cas9-mediated mutagenesis combined with a dual sgRNAs strategy, we generated the *B*. *mori Clk*^*Δ29*^, which stably inherits a non-functional *Clk* allele. Our characterisation of the *Clk*^*Δ29*^ mutants demonstrated the central role of *Clk* in *B*. *mori* circadian rhythmicity at molecular and behavioural levels. CLK appeared to modulate physiological processes involved in *B*. *mori* development and growth, as two independently generated *Clk*^*-*^ mutant strains (*Clk*^*Δ29*^ and *Clk*^*Δ1922*^) exhibited prolonged developmental times during the fifth instar and increased pupal and silk shell weights. Additionally, on a mulberry leaf diet, the non-functional CLK phenotype increased silk yield, the most important economic parameter of sericulture, by up to 7%, and enhanced overall pupal weight by up to 25%. Integrating these findings with recent studies underscores the potential of genetic engineering for the enhancement of sericultural traits in *B*. *mori*. However, further analyses are needed to determine whether our observed increases are due to clock-dependent or clock-independent functions of CLK. Elucidating the specific molecular mechanisms regulating these phenotypes could further enhance the commercial potential of the domestic silkworm.

## Supporting information

S1 Table Single guide RNAs designed for *B*. *mori Clk* mutagenesis. (DOCX)

S2 Table Primers used in CRISPR/Cas9 mutagenesis screening and qPCR experiments. (DOCX)

S1 Raw images Gel images originating Fig 1C and 1D. (PDF)

S1 File qPCR data. (XLSX)

S2 File Eclosion data. (XLSX)

S3 File Hatching data. (XLSX)

S4 File 5th larval weight data. (XLSX)

S5 File ClkD29 Silk and pupae data_artificial diet. (XLSX)

S6 File ClkD1922 Silk and pupae data_artificial diet. (XLSX)

S7 File ClkD29Silk and pupae data_mulberry leaves. (XLSX)
