# Correction: Enhanced silk production and pupal weight in *Bombyx mori* through CRISPR/Cas9-mediated circadian *Clock* gene disruption

**DOI:** 10.1371/journal.pone.0322240

**Published:** 2025-04-04

**Authors:** The PLOS One Staff

There is an error in affiliation 2 for author Daniel J. Brady. The correct affiliation 2 is: Fraunhofer Institute for Molecular Biology and Applied Ecology—IME, Branch for Bioresources, Gießen, Germany.

The publisher apologizes for the error.
