# Supplementary material for: Enhanced silk production and pupal weight in Bombyx mori through CRISPR/Cas9-mediated circadian Clock gene disruption
Source: PLoS One. 2025 Jan 27;20(1):e0317572. doi: 10.1371/journal.pone.0317572 (PMC11771929; doi:10.1371/journal.pone.0317572)
Supplement: S1 Table — (DOCX) [file pone.0317572.s001.docx]

**Table S1. Single guide RNAs designed for *B. mori* *Clk* mutagenesis.**

| **sgRNA** | ***Clk* exon** | **DNA strand** | **target sequence (5'-3')** | **PAM** | **% GC** |
| --- | --- | --- | --- | --- | --- |
| 1 | 2 | + | CCAAACAGTATGGAAGACGA | CGG | 50 |
| 2 | 3 | + | CTTGAGTGAAAAAAAGCGAC | GGG | 55 |
