# Supplementary material for: Enhanced silk production and pupal weight in Bombyx mori through CRISPR/Cas9-mediated circadian Clock gene disruption
Source: PLoS One. 2025 Jan 27;20(1):e0317572. doi: 10.1371/journal.pone.0317572 (PMC11771929; doi:10.1371/journal.pone.0317572)
Supplement: S2 Table — (DOCX) [file pone.0317572.s002.docx]

**Table S2. Primers used in CRISPR/Cas9 mutagenesis screening and qPCR experiments.**

| **Gene** | **Accession ID** | **Experiment type** | **Primer name** | **Sequence (5'-3')** | **Size (bp)** |
| --- | --- | --- | --- | --- | --- |
| *Clk* | XM_038011384.1 | sgRNA *in vitro* evaluation and screening | BmClk_For | AAATATACCAGAAAGACTGTGCTG | 1372 |
|  |  |  | BmClk_Rev | TGTTGTGATTTGAAGTGGC |  |
|  |  | qPCR | BmClk_qPCR_ For | ACACAAATACCCCGAATCCCA | 171 |
|  |  |  | BmClk_qPCR_ Rev | CTTGTTGATATCCGGACGAATCA |  |
| *-* | pJET plasmid | Sequencing | pJET1-2F | CGACTCACTATAGGGAGAGCGGC |  |
| *cyc* | NM_001043517.1 | qPCR | Bmcyc_F | CAACAACTACAACCACGGCG | 127 |
|  |  |  | Bmcyc_R | ATCGGTATCATGGCGCTCAG |  |
| *per* | DQ393413.1 | qPCR | Bmper_F | GTTGGGTAACACCGAGGAG | 237 |
|  |  |  | Bmper_R | CGCTGGAATATGGTGATCGG |  |
| *tim* | DQ392962.1 | qPCR | Bmtim_F | TCAGTGGGAACATCACCGAC | 117 |
|  |  |  | Bmtim_R | ACGCGGACGTTTTAAGTTGC |  |
| *rp49* | AY769302.1 | qPCR | rp49_F | GTTCCAACAAGAAGACCCG | 159 |
|  |  |  | rp49_R | TCCACGATCAGCTTCCGCTT |  |
| *Act3* | NM_001126254.1 | qPCR | Act3_F | GACTTGGCCGGTCGTGACC | 94 |
|  |  |  | Act3_R | GAACGATTTCCCGCTCGGCAG |  |
