# Supplementary figures and images for: Enhanced silk production and pupal weight in Bombyx mori through CRISPR/Cas9-mediated circadian Clock gene disruption
Source: PLoS One. 2025 Jan 27;20(1):e0317572. doi: 10.1371/journal.pone.0317572 (PMC11771929; doi:10.1371/journal.pone.0317572)

Original gel image originating Fig1 C

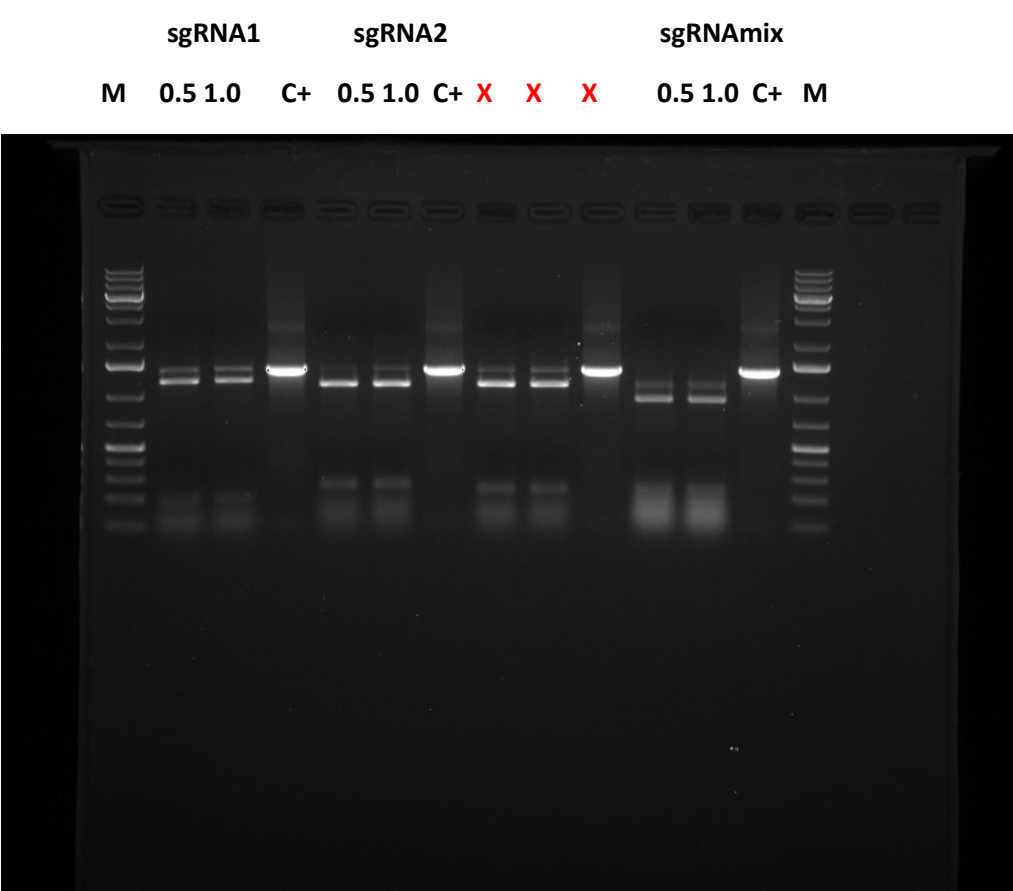

Original gel image originating Fig1 D

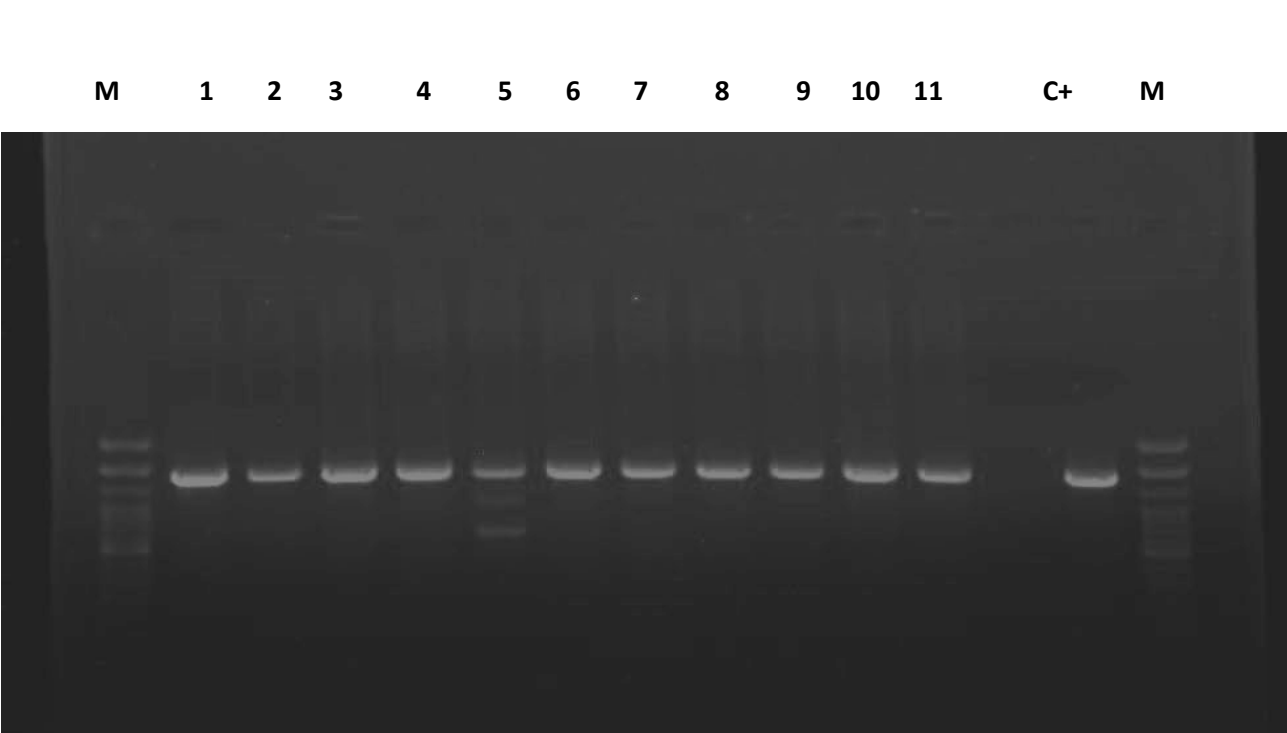

Supplement: S1 Raw images — (PDF) [file pone.0317572.s003.pdf]
